# Investigating the reasons behind a later or missed diagnosis of attention‐deficit/hyperactivity disorder in young people: A population cohort study

**DOI:** 10.1002/jcv2.12301

**Published:** 2024-12-18

**Authors:** Isabella Barclay, Kapil Sayal, Tamsin Ford, Ann John, Mark J. Taylor, Anita Thapar, Kate Langley, Joanna Martin

**Affiliations:** ^1^ Centre for Neuropsychiatric Genetics and Genomics Cardiff University Cardiff UK; ^2^ Wolfson Centre for Young People's Mental Health Cardiff University Cardiff UK; ^3^ School of Medicine, Institute of Mental Health University of Nottingham Nottingham UK; ^4^ Department of Psychiatry University of Cambridge Cambridge UK; ^5^ Swansea University Medical School Wales UK; ^6^ Department of Medical Epidemiology and Biostatistics Karolinska Institutet Stockholm Sweden; ^7^ School of Psychology Cardiff University Cardiff UK

**Keywords:** ADHD, emotional dysregulation, missed diagnosis, sex differences

## Abstract

**Background:**

Attention Deficit Hyperactivity Disorder (ADHD) is a common neurodevelopmental condition, more often diagnosed in males. In many individuals, particularly females, ADHD is diagnosed later or missed, the reasons for this are not fully understood. Timely diagnosis is needed to provide support, management, and treatment to improve outcomes. This study aimed to understand why some young people with ADHD experience later or missed diagnosis and to consider sex differences.

**Methods:**

This study included 9991 (females = 43.69%) individuals from the Millenium Cohort Study, a UK based population study which defined recognised ADHD by a parent‐reported clinician diagnosis, and unrecognised ADHD by parent‐reported questionnaires. Behavioural and emotional difficulties, engagement in leisure activities, and parental characteristics, were compared between those recognised earlier (by ages 5/7, *n* = 264, *f* = 19.3%) versus later (by ages 11/14, *n* = 260, *f* = 21.2%), and those recognised (*n* = 524, *f* = 20.2%) versus unrecognised (*n* = 1,138, *f* = 38.7%) using logistic regression, with odds ratios (OR) and 95% confidence intervals (CI) analysed. Sex differences were investigated with an interaction analysis.

**Results:**

Those recognised with ADHD earlier had more peer, conduct, and emotional problems, emotional dysregulation, lower cognitive ability, and poorer prosocial skills compared with those recognised later, ORs ranged from 0.27 (95% CI = 018, 0.41) to 1.20 (95% CI = 1.20, 1.32). Similar findings were seen when comparing those with recognised and unrecognised ADHD; ORs ranged from 0.11 (95% CI = 0.09, 0.15) to 1.31 (95% CI = 1.19, 1.43). Additionally, those recognised were more likely to have diagnosed autism and have less reported physical activity. Sex stratification showed that recognised males had higher emotional dysregulation than unrecognised males, but this was not seen in females.

**Conclusions:**

Our findings highlight the need to consider ADHD referral, regardless of cognitive and prosocial ability or comorbidities, if children are displaying ADHD symptoms. Additionally, symptoms of ADHD not traditionally included in screening criteria, such as emotional dysregulation, should be considered to improve gender‐inclusive recognition of ADHD.

**Key Points**

- Undiagnosed Attention Deficit Hyperactivity Disorder (ADHD) is untreated ADHD, which is likely to undermine development, particularly in the academic and social domains.
- ADHD is known to be diagnosed later, and less often, in females.
- This study showed that children were less likely to be diagnosed with ADHD if they have higher cognitive ability, more physical activity, more prosocial skills, no diagnosis of autism, and fewer behavioural, emotional, peer and conduct issues.
- Males were more likely to be diagnosed if they have more emotional dysregulation, this was not the case for females.
- All children with elevated ADHD symptoms and associated impairment should be considered for further ADHD assessment, regardless of sex, cognitive ability, prosocial skills or emotional and disruptive behaviours.

## INTRODUCTION

Attention Deficit Hyperactivity Disorder (ADHD) is a common neurodevelopmental condition that affects people of any age, sex,^1^ gender, or ethnicity. ADHD diagnosis is important, as unrecognised ADHD likely means untreated ADHD, which can have negative impacts psychologically, financially, academically and socially (Hamed et al., 2015). ADHD, even treated, is associated with many negative life outcomes, including mental health conditions for example, anxiety, depression, eating disorders; physical health conditions, substance use disorder, incarceration, and self‐harm (Biederman et al., 2007; Gnanavel et al., 2019; Huntley et al., 2012; Katzman et al., 2017; Levin & Rawana, 2016; Meza et al., 2021; Young et al., 2015). Despite the importance of a timely diagnosis, many people with ADHD are not diagnosed.

Sex ratios for diagnosis vary between 1.9:1 and 10:1, depending on sample size, type and age; however, a consistent observation is that many more males are diagnosed than females in childhood (Faraone et al., 2015; Gaub & Carlson, 1997; Mowlem, Rosenqvist, et al., 2019; Sandberg, 2002; Slobodin & Davidovitch, 2019; Willcutt, 2012). Once thought of as purely a sex bias in prevalence, it is now understood that there is also a sex bias in diagnosis, driven by missed and late diagnoses in females (Bruchmüller et al., 2012; Martin et al., 2024). The ADHD diagnostic criteria were developed based on characteristics typically presented by young males, meaning other aspects strongly linked to ADHD, like emotional symptoms, which may be affected by sex, both in occurrence and perception, are not part of the current diagnostic criteria (Faraone et al., 2019).

Various explanations have been proposed for this sex bias. Females may be more likely to have inattentive ADHD symptoms which could be missed by others, are more likely than males to have a co‐occurring mental health condition that could overshadow ADHD, and professionals have been shown to be less likely to diagnose ADHD in females (Bruchmüller et al., 2012; Gershon, 2002; Lahey et al., 1994; Morgan, 2023; Rucklidge, 2010; Schuck et al., 2019; Skoglund et al., n.d.; Waite, 2007). Any child or young person may have a diagnosis delayed by mitigating factors such as parental scaffolding, cognitive ability, high levels of physical activity, or overshadowing from mental health symptoms and alternative diagnoses (Cadenas et al., 2020; Cerrillo‐Urbina et al., 2015; Taylor et al., 2019). The latter is particularly important when considering co‐occurring autism, as until the Diagnostic and Statistical Manual of Mental Disorders (DSM‐5) in 2013, autism was considered an exclusion criteria for ADHD diagnosis, due to the known overlap of the two conditions, this may have led to a delayed diagnosis in children with both (Leitner, 2014; Rong et al., 2021). Alternative diagnosis is even more concerning when considering unconscious bias, with studies showing African‐American and Latino children are more likely to receive a diagnosis of a disruptive behaviour disorder than ADHD when compared with their non‐Hispanic white peers (Fadus et al., 2020; Mandell et al., 2007). Lastly, children displaying non‐disruptive symptoms in the classroom are less likely to be assessed for ADHD than their disruptive peers (Felt et al., 2014; Mowlem, Agnew‐Blais, et al., 2019).

There is ongoing debate in the literature about whether late diagnosed ADHD could be explained by late‐onset (i.e., ADHD symptoms that develop after the age of 12) (Riglin et al., 2022; Taylor et al., 2019). These studies describe adults seemingly first beginning to express ADHD symptoms in adulthood, and therefore only being diagnosed after childhood. However, these groups are more likely to have compensatory factors (e.g., higher parental education, cognitive ability, and family income) protecting them from negative outcomes, or have alternative diagnoses that may halt exploration for further conditions such as ADHD (Riglin et al., 2022; Taylor et al., 2019).

### Research questions

The reasons why some children have a later diagnosis of ADHD are poorly understood. This study compares individuals with ADHD recognised earlier in childhood to those recognised later, to examine any factors (e.g., emotional and behavioural difficulties) that may contribute to diagnosis timing. Additionally, the study compared those with clinically recognised ADHD in childhood to those who had probable but unrecognised ADHD.

We hypothesised that several factors may reduce the likelihood of receiving an ADHD diagnosis, including: fewer behavioural or emotional difficulties, better prosocial skills, higher cognitive ability, greater engagement in hobbies, and higher physical activity. We also performed an exploratory sex‐stratified analysis.

## MATERIALS AND METHODS

### Data

This study used data from the Millennium Cohort Study (MCS) (Joshi & Fitzsimons, 2016). This is a prospective, longitudinal birth cohort study in the UK. These data were collected from 19,483 UK residents born between September 2000 and January 2002, at 9 months, and then at 3, 5, 7, 11, 14, and 17 years. Everyone who was born during this time‐frame and still alive at 9 months was eligible for inclusion. The cohort design was clustered and stratified with oversampling to ensure children living in areas of the UK with high poverty or large ethnic minority populations were adequately represented (Joshi & Fitzsimons, 2016). Data were collected during home visits, completed by trained interviewers with computer assistance. At each data collection stage the study was reviewed and approved by relevant research ethics committees. Data were provided with written parental informed consent, and then at age 11 the child also provided assent. The ethical approval for this specific study was granted by the Cardiff University School of Medicine Research Ethics Committee (reference SMREC 22/48). Data from the following timepoints were used in this study: 5 (*n* = 15,460), 7 (*n* = 14,043), 11 (*n* = 13,469), and 14 years (*n* = 11,872).

### Inclusion criteria

**FIGURE 1:**
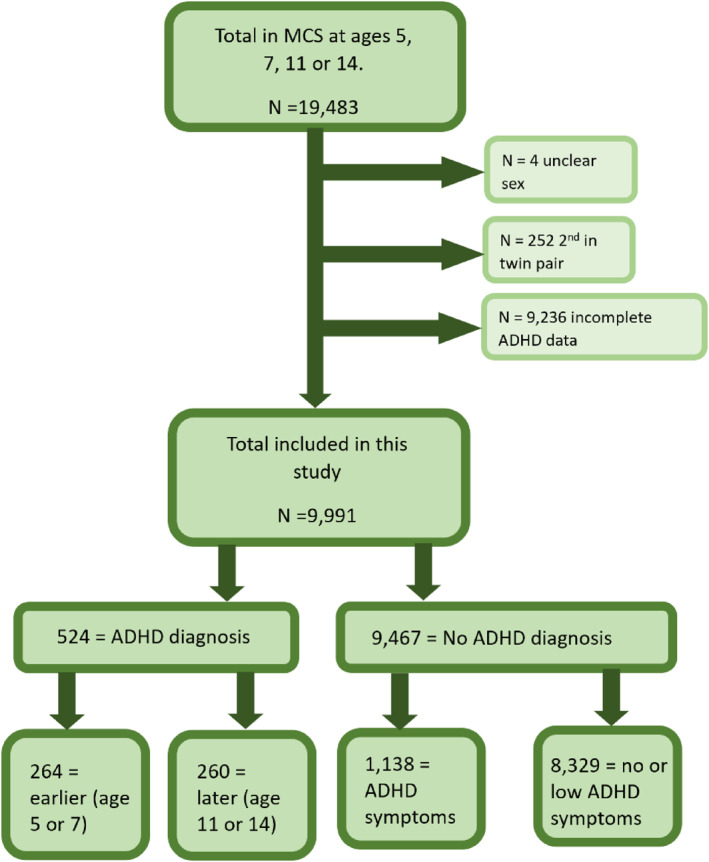
Flow chart of inclusions in this study. ADHD, Attention Deficit Hyperactivity Disorder; MCS, Millennium Cohort Study; *N*, sample size.

To be included, there needed to be clear assigned sex at birth data (*n* = 4 excluded), and complete ADHD data (where they answered ‘yes’ at least once to the question ‘has your child been diagnosed with ADHD’, or ‘no’ at every time point (*n* = 9236 excluded). Those excluded were more likely to have lower maternal education, to not own the house they were living in, be below the poverty line, and have a lower maternal age at birth). One from each twin pair were excluded (*n* = 252 excluded, of these, 9 had diagnosed ADHD). See Figure 1 for a flowchart of inclusions.

### Measures

#### ADHD

Parents/carers were asked ‘Has a doctor or health professional ever told you that [^Cohort child's name] had any of the following problems?… ADHD?’ when the child was 5, 7, 11 and 14 years old. If they answered ‘yes’ at any time point, the child was recorded as having clinically recognised ADHD; if ‘yes’ at the age 5 or 7 timepoint, they were considered to have an earlier diagnosis; and if ‘yes’ at age 11 or 14, but not before, they were considered to have a later diagnosis.

We used the parent‐reported Strengths and Difficulties Questionnaire (SDQ) hyperactivity subscale to assess possible ADHD, which includes hyperactive‐impulsive and inattentive symptoms. Parents completed the SDQ at ages 5, 7, 11 and 14. This measure, and cut‐points, have been shown to be a reliable measure of ADHD symptoms with good specificity (Goodman, 2001). Question responses were derived to make an overall score, ranging between 0 and 10. The cut‐point for a ‘high’ ADHD symptom score was 7 or above (Green et al., 2005). Children with a high SDQ hyperactivity score and therefore possible ADHD, but no clinical diagnosis of ADHD at any timepoint (up to age 14) were defined as having ‘unrecognised ADHD’.

At ages 5 and 7, parents were asked about the impact of the ADHD symptoms including which areas the symptoms were in, how long they had been present, whether they were upsetting for the child and whether they interfered with everyday life, using the SDQ Impact Supplement. If the impact score was between 2 and 10, impact from symptoms was considered present (Green et al., 2005). Children with high SDQ‐hyperactivity and impact scores at age 5 or 7 also with impact present at either age 5 or 7, but no clinical diagnosis of ADHD by age 14, were defined as having ‘unrecognised ADHD with impact’. Impact information was not available at ages 11 or 14.

Children were considered to have no ADHD if they had no or low symptoms (score <7) on the SDQ‐hyperactivity scale and their parents/carers answered no to the question ‘has your child been diagnosed with ADHD’ at all time points.

#### Mental health, behaviour and activities

Parent‐reported SDQ scores for conduct problems, emotional problems, peer relationships and prosocial skills, at ages 5 and 7 were analysed (Goodman, 1997). Emotional dysregulation was assessed using the parent‐reported Child Social and Behavioural Questionnaire at ages 5 and 7, adapted from the Adaptive Social Behaviour Inventory as used in other published MCS studies (Antony et al., 2022). This included the following questions: ‘child shows wide mood swings’, ‘child gets over excited’, ‘child is easily frustrated’, ‘child quickly gets over being upset’, ‘child is impulsive and acts without thinking’, where the answer options were: ‘not true’, ‘somewhat true’, ‘certainly true’ and ‘can't say’. Scores were derived to give a mean score between 1 and 3, with higher scores indicating greater difficulties. A child was considered autistic if their parents had ever answered ‘yes’ to the question: ‘Has a doctor or health professional ever told you that [^Cohort child's name] had any of the following problems? … Autism or Asperger's Syndrome’ across ages 5, 7, 11 and 14.

Physical activity at age 7 was based on the parent‐reported question: ‘on average, how many days per week does [child] usually go to a club or class to do sport, or any other physical activity like swimming, gymnastic, football, dancing etc.’, scored between 0 and 5 as a continuous variable (where 0 = 0 days per week and 5 = 5+ days per week). A binary variable was derived for hobby frequency, including self‐reported answers to the following questions asked at age 11: ‘how often do you draw, paint or make things whilst not at school’ and ‘how often do you read for enjoyment whilst not at school?’, where the answer was considered as high/often if they answered ‘most days’ to either question.

#### Cognitive ability

The British Ability Scale is a validated tool which was used to measure cognitive ability at age 5 (Elliott et al., 1996; Hill, 2005). As recommended, a principal component analysis was run on the *t*‐scores of the three scales (picture recognition, pattern similarity and naming vocabulary), and the first principal component was extracted and used for analysis (Connelly, 2013).

#### Parental characteristics

Parental characteristics were measured to assess familial impact on ADHD recognition. Parental education was calculated using reported educational achievement the first response female parent had achieved by the time their child was aged 7. A binary variable was created, with those who had achieved a higher education award and those who had not. Parental depression/anxiety was indicated by the first‐respondent parent's answer to the question: ‘have you received a diagnosis of anxiety/depression from your doctor’; if they answered yes at any timepoint (9 months to 14 years) then they were considered to have depression/anxiety.

### Statistical analysis

To address the study aims, two main comparisons were made. The first compared those with an earlier diagnosis of ADHD, by the age 5 or 7 (coded as 0), against those who were diagnosed with ADHD later, by the age 11 or 14 but not before (coded as 1). These two groups were considered to have recognised ADHD. This group was not split by sex, due to small numbers of females.

The second analysis compared those with diagnosed ADHD at any age (coded as 0), considered the ‘recognised’ ADHD group, to those with a high SDQ‐hyperactivity score but no diagnosis of ADHD between ages 5–14 years (coded as 1), considered the ‘unrecognised ADHD’ group. This comparison was then stratified by sex, the results of which were then compared using an interaction test. Finally, a sub‐group of the unrecognised ADHD group who also had a high impact score at ages 5 or 7 (coded as 1), was compared against those who had recognised ADHD at any age (coded as 0).

Logistic regression analysis in R (RStudio 2023.06.1 + 524) was used for all analyses, with variables of interest as the predictors and the grouping variable as the outcome. Exact age (in days at ages 5 and 7, and to the nearest 10th of the year at age 11) when data collection occurred was included as a covariate in all analyses. Analyses were corrected for multiple testing using the False Discovery Rate (FDR).

Two sensitivity tests were run. Test one excluded children who had no response data for the question ‘has your child been diagnosed with ADHD’ at ages 5 and 7, but did at ages 11 and 14, to test whether this missing data had an impact on the results. Test two redefined the groups for earlier and later diagnosis, comparing those with a diagnosis at age 5, 7 or 11 with those who had a first reported diagnosis at age 14, to test whether the definition of earlier versus later in this study affected the results.

Where differences were identified in the sex stratified analyses, logistic regression analyses were undertaken to explore whether such differences were present only in those with ADHD symptoms, or if they were reflective of sex differences across the sample.

### Lived experience consultation

A Youth Advisory Group of six individuals between 14 and 24 years old with lived experiences of neurodevelopmental conditions (e.g., ADHD, autism) were consulted during development of this study. They gave suggestions for factors to be investigated, and gave opinions on the planned comparisons, which we took on board.

## RESULTS

### Sample description

There were 9991 participants who met study inclusion criteria (see Figure 1). Of these, 264 (2.6% of the analysed study sample, 50.4% of the ADHD sample, females = 51) were diagnosed with ADHD at age 5 or 7, and 260 (2.6% of the analysed study sample, 49.6% of the ADHD sample, females = 55) were diagnosed at age 11 or 14.

There were 1138 individuals (11.4% of the analysed study sample, females = 440) who had unrecognised ADHD across ages 5, 7, 11 and 14, and 215 (2.2% of the analysed study sample, 18.9% of the total unrecognised ADHD group, females = 64) of them had high symptoms and a high impact score at age 5 or 7. In the earlier recognised group, there was a male to female ratio of 4.2:1, in the later recognised group it was 3.7:1, in the unrecognised group 1.6:1, and in the unrecognised with impact group 2.4:1. There were 8329 children with no or low ADHD symptoms and no diagnosis.

The unrecognised and recognised ADHD groups had higher mean SDQ‐hyperactivity, and impact scores than the no/low ADHD group. For all but one measure (age 14 SDQ‐hyperactivity), the hyperactivity and impact scores were higher in the earlier recognised group, compared to the later recognised.

Details of characteristics, demographics, and ADHD scores can be seen in Tables 1 and 2.

**TABLE 1:** Child and parent characteristics and socioeconomic characteristics.

	Recognised ADHD		Unrecognised ADHD		No ADHD
	Earlier (ages 5–7) *n* = 264	Later (ages 11–14) *n* = 260	High SDQ symptoms (ages 5–14) *n* = 1138	High SDQ symptoms and impact (ages 5–7) *n* = 215	No ADHD diagnosis and low SDQ symptoms *n* = 7233
Sex (female) *n* (%)	51 (19.3%)	55 (21.2%)	440 (38.7%)	64 (29.8%)	3858 (53.3%)
Ethnicity *n* (%)					
White	234 (88.6%)	229 (88.1%)	947 (83.2%)	190 (88.4%)	6380 (88.2%)
Mixed	9 (3.4%)	13 (5.0%)	31 (2.7%)	5 (2.3%)	182 (2.5%)
South Asian	14 (5.3%)	8 (3.1%)	118 (10.4%)	12 (5.6%)	448 (6.2%)
Other ethnic group (incl Black Caribbean, Black African, and Chinese)	7 (2.7%)	9 (3.5%)	42 (3.7%)	8 (3.7%)	226 (3.1%)
Relative low income/below 60% median poverty indicator *n* (%)	139 (52.7%)	114 (43.9%)	459 (40.3%)	82 (38.1%)	1676 (23.2%)
Housing tenure *n* (%)					
Homeowner	96 (36.4%)	95 (36.5%)	651 (57.2%)	125 (58.1%)	5521 (76.3%)
Other (social housing, private rent etc.)	141 (53.4%)	122 (46.9%)	484 (42.5%)	90 (41.9%)	1685 (23.3%)
Birth weight (KG), mean (SE)	3.29 (0.030)	3.33 (0.039)	3.31 (0.019)	3.27 (0.047)	3.39 (0.007)
Maternal age at childbirth, mean (SEM)	26.3 (0.38)	26.7 (0.38)	27.8 (0.17)	27.9 (0.37)	29.73 (0.06)

Abbreviations: ADHD, Attention Deficit Hyperactivity Disorder; SDQ, Strengths and Difficulties Questionnaire; SEM, standard error of the mean.

**TABLE 2:** Descriptive ADHD statistics: mean and standard error of the mean (SEM) SDQ hyperactivity and impact scores.

	Recognised ADHD		Unrecognised ADHD		No ADHD
	Earlier (aged 5–7) *n* = 264	Later (aged 11–14) *n* = 260	High SDQ symptoms *n* = 1138	High SDQ symptoms and impact *n* = 215	No ADHD diagnosis and low SDQ symptoms *n* = 7233
SDQ hyperactivity mean (SEM)					
Age 5	7.12 (0.17)	5.26 (0.17)	6.30 (0.07)	7.25 (0.15)	2.55 (0.02)
Age 7	7.77 (0.16)	6.21 (0.19)	6.73 (0.07)	8.28 (0.11)	2.53 (0.02)
Age 11	6.97 (0.20)	6.88 (0.18)	6.29 (0.07)	6.79 (0.15)	2.34 (0.02)
Age 14	6.36 (0.22)	7.11 (0.18)	5.85 (0.07)	5.75 (0.17)	2.27 (0.02)
SDQ impact mean (SEM)					
Age 5	2.30 (0.18)	0.76 (0.12)	0.56 (0.04)	1.98 (0.16)	0.02 (0.001)
Age 7	3.25 (0.20)	1.38 (0.15)	0.92 (0.05)	3.29 (0.16)	0.03 (0.002)

Abbreviations: ADHD, Attention Deficit Hyperactivity Disorder; SDQ, Strengths and Difficulties Questionnaire.

### Comparison of earlier versus later recognised ADHD

**FIGURE 2:**
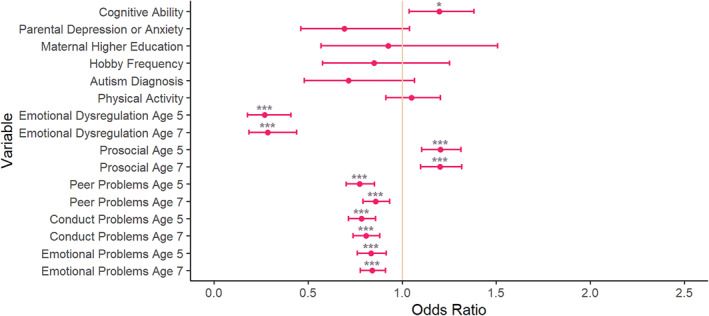
Comparison of earlier recognised ADHD versus later recognised ADHD. Variables were explored to understand their relationship with the timing of ADHD diagnosis. With exact age as a covariate. *** *p* < 0.001, ** *p* < 0.01 and * *p* < 0.05, after FDR correction. Where earlier recognised ADHD is coded as 0, and later recognised ADHD is coded as 1. ADHD, Attention Deficit Hyperactivity Disorder; FDR, False Discovery Rate; SDQ, Strengths and Difficulties Questionnaire.

As shown in Figure 2 and Table S1, after FDR correction, individuals with a later diagnosis of ADHD were more likely to have a higher cognitive ability (odds ratios [OR] = 1.20, 95% confidence intervals [CI] (1.04, 1.38)) and better prosocial skills at age 5 (OR = 1.20, 95% CI (1.10, 1.31)) and 7 (OR = 1.20, 95% CI (1.10, 1.32)) than those who were diagnosed earlier.

Those with a later diagnosis of ADHD had lower levels of emotional dysregulation at ages 5 (OR = 0.27, 95% CI (0.18, 0.41)) and 7 (OR = 0.29, 95% CI (0.19, 0.44)), peer problems at 5 (OR = 0.77, 95% CI (0.70, 0.85)) and 7 (OR = 0.86, 95% CI (0.79, 0.93)), conduct problems at 5 (OR = 0.78, 95% CI (0.71, 0.86)) and 7 (OR = 0.81, 95% CI (0.74–0.88)), and emotional problems at age 5 (OR = 0.84, 95% CI (0.76, 0.92)) and 7 (OR = 0.84, 95% CI (0.78, 0.91)), compared to those who were diagnosed with ADHD earlier.

Some variables showed no strong difference between the earlier and later diagnosed ADHD groups, including hobby frequency (OR = 0.85, 95% CI (0.58, 1.25)), autism diagnosis (OR = 0.71, 95% CI (0.48, 1.07)), physical activity (OR = 1.05, 95% CI (0.91, 1.20)), parental depression/anxiety (OR = 0.69, 95% CI (0.46, 1.04)), and maternal higher education (OR = 0.93, 95% CI (0.57, 1.51)).

### Comparison of recognised versus unrecognised ADHD

**FIGURE 3:**
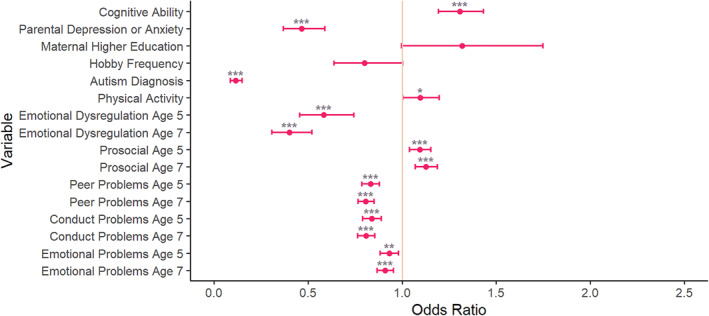
Comparison of those with recognised ADHD versus those with unrecognised ADHD. Variables were explored to understand their relationship with the likelihood of ADHD diagnosis. With exact age as a covariate. *** *p* < 0.001, ** *p* < 0.01 and * *p* < 0.05, after FDR correction. Where recognised ADHD is coded as 0, and unrecognised ADHD is coded as 1. ADHD, Attention Deficit Hyperactivity Disorder; FDR, False Discovery Rate; SDQ, Strengths and Difficulties Questionnaire.

As shown in Figure 3 and Table S2, after FDR correction, those with unrecognised ADHD were more likely to have a higher cognitive ability (OR = 1.31, 95% CI (1.19, 1.43)), have better prosocial skills at ages 5 (OR = 1.09, 95% CI (1.04, 1.15)) and 7 (OR = 1.13, 95% CI (1.07, 1.19)), and reported more physical activity (OR = 1.10, 95% CI (1.00, 1.20)) than those with a recognised diagnosis.

They were also less likely to have a parent with depression/anxiety (OR = 0.47, 95% CI (0.37, 0.59)) or a diagnosis of autism (OR = 0.11, 95% CI (0.09, 0.15)) than those with recognised ADHD. They also had lower levels of emotional dysregulation at ages 5 (OR = 0.58, 95% CI (0.46, 0.74)) and 7 (OR = 0.40, 95% CI (0.31, 0.52)), peer problems at ages 5 (OR = 0.83, 95% CI (0.79, 0.88)) and 7 (OR = 0.81, 95% CI (0.77, 0.85)), conduct problems at age 5 (OR = 0.84, 95% CI (0.79, 0.89)) and 7 (OR = 0.81, 95% CI (0.76, 0.85)) and emotional problems at age 5 (OR = 0.93, 95% CI (0.88, 0.98)) and 7 (OR = 0.91, 95% CI (0.87, 0.95)).

The remaining variables showed no strong difference between the recognised and unrecognised ADHD groups, including hobby frequency (OR = 0.80, 95% CI (0.64, 1.00)) and maternal higher education (OR = 1.32, 95% CI (1.00, 1.75)).

### Sex stratified analyses

After FDR, females with unrecognised ADHD were less likely to have a diagnosis of autism (OR = 0.16, 95% CI (0.09, 0.28)) and less likely to have peer problems at age 7 (OR = 0.81, 95% CI (0.73, 0.91)) than females with recognised ADHD, as shown in Table S3. The males showed a similar pattern of results to the full sample analysis, with the addition that higher level of maternal education was more likely in the unrecognised group (OR = 1.54, 95% CI (1.10, 2.15)), as shown in Table S4.

**FIGURE 4:**
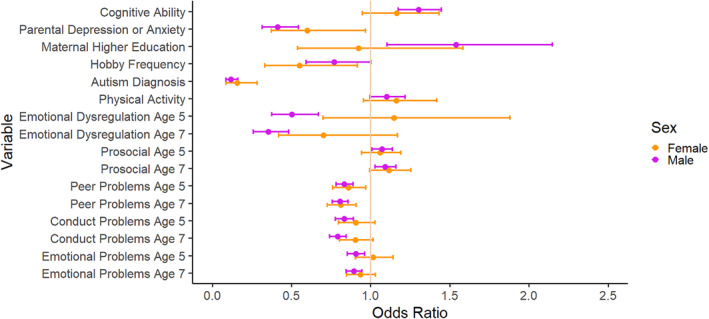
Comparing those with recognised ADHD versus those with unrecognised ADHD stratified by sex. Variables were explored to understand their relationship with the likelihood of ADHD diagnosis, here the results of the male and female split analysis were compared in order to analyse differences and similarities. With exact age as a covariate. *p*‐values are shown in Supporting Information S1. Where recognised ADHD males versus unrecognised ADHD males is coded as 0, and recognised ADHD females versus unrecognised ADHD females is coded as 1. ADHD, Attention Deficit Hyperactivity Disorder; SDQ, Strengths and Difficulties Questionnaire.

When comparing the results with interaction analysis, shown in Figure 4 and Table S5, the results of males and females were very similar, except for emotional dysregulation, which showed a significant difference in effect sizes for males and females at ages 5 (OR = 2.27, 95% CI (1.28, 4.02)) and 7 (OR = 1.97, 95% CI (1.08, 3.58)). While recognised males were more likely to have higher levels of emotional dysregulation than unrecognised males, recognised and unrecognised females had similar levels of emotional dysregulation.

### Comparison of those with recognised ADHD and those with unrecognised ADHD and impact

**FIGURE 5:**
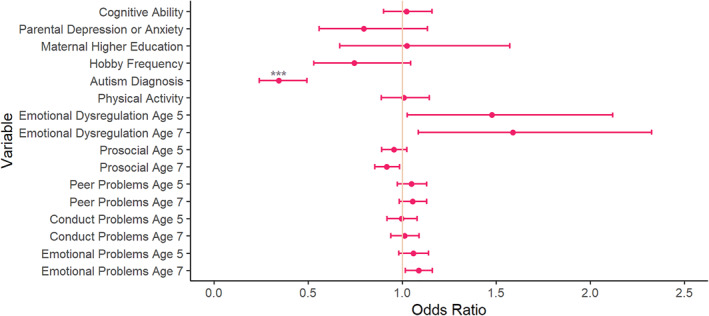
Comparison of those with recognised ADHD versus those with unrecognised ADHD and impact. Variables were explored to understand their relationship with the likelihood of ADHD diagnosis. With exact age as a covariate. *** *p* < 0.001, ** *p* < 0.01 and * *p* < 0.05, after FDR correction. Where recognised ADHD is coded as 0, and unrecognised ADHD and impact is coded as 1. ADHD, Attention Deficit Hyperactivity Disorder; FDR, False Discovery Rate; SDQ, Strengths and Difficulties Questionnaire.

As shown in Figure 5 and Table S6, after FDR correction those who had recognised ADHD were more likely to have a diagnosis of autism (OR = 0.34, 95% CI (0.24, 0.49)) than those with unrecognised ADHD with impact. The remainder of the variables showed no strong difference between the two groups (*p* < 0.05) after FDR correction.

### Sensitivity analysis

Excluding individuals with missing ADHD data at age 5 and 7, generated similar results to the main analysis except that a diagnosis of autism was more likely in the early diagnosed group (OR = 0.66, 95% CI (0.44, 0.98)) as was having a parent with depression/anxiety (OR = 0.63, 95% CI (0.42, 0.95)) where these variables previously did not differ between the groups (see Table S7). When we redefined the earlier (ages 5, 7 and 11, *N* = 426) and later (age 14, *N* = 98) diagnosis groups, the results were also similar to the main analysis except that cognitive ability was no longer different between the two groups (OR = 1.17, 95% CI (0.97, 1.42)) (see Table S8).

### Post‐hoc analysis

We examined differences in emotional dysregulation between females with recognised and unrecognised ADHD, compared to females without ADHD. Levels of emotional dysregulation were significantly higher in the recognised and unrecognised female ADHD groups at age 5 (Recognised ADHD: OR = 9.13, 95% CI (5.77, 14.44), *p* < 0.0001. Unrecognised ADHD: OR = 10.84, 95% CI (8.51, 13.82), *p* < 0.0001) and age 7 (Recognised ADHD: OR = 18.56, 95% CI (11.45, 30.09), *p* < 0.0001. Unrecognised ADHD: OR = 14.43, 95% CI (11.19, 18.59), *p* < 0.0001).

## DISCUSSION

This study investigated factors contributing to earlier recognition of ADHD, and why some children go unrecognised despite having ADHD symptoms and impact. Interestingly, the male to female ratio was higher in both the earlier (4.2:1) and later (3.7:1) recognised groups compared to the unrecognised group (1.6:1), suggesting relatively more males are being recognised. The results suggested that children with more emotional and behavioural difficulties, greater emotional dysregulation, lower cognitive ability and poorer prosocial skills were more likely to receive an earlier diagnosis. Findings were similar when studying those with recognised versus unrecognised ADHD, with the addition of parental depression/anxiety, lower physical activity levels and an autism diagnosis being more common in the recognised group. Sex stratification showed that emotional dysregulation was a key differentiating factor between those with and without recognised ADHD in males, but not females. When comparing those with recognised and unrecognised ADHD with impact, the only difference was those recognised were more likely to have diagnosed autism.

Those with earlier recognised ADHD were more likely to have emotional, social and behavioural difficulties, which may cause parental or teacher concern due to disruption concerns in the classroom leading to further investigation (Felt et al., 2014). Indeed, previous studies suggest that the impact of difficulties on others around the child, and comorbidity predict referral to specialist services for psychiatric disorders in general (Ford et al., 2008; Sayal et al., 2006). Indeed, in our study, the final comparison comparing recognised ADHD to unrecognised ADHD with high reported levels of impact, the only differing factor was an increased likelihood of an autism diagnosis in the recognised group. Evidently, children with symptoms and impact might benefit from an ADHD assessment, and this finding suggests that the burden of multiple difficulties may increase likelihood of contact with specialist services. Additionally, it may be that during assessment for one, another condition is highlighted, particularly for ADHD and autism, where they are often found to co‐occur, and those with both are more likely to struggle with functioning and daily life (Cooper et al., 2014; Davis & Kollins, 2012; Rao & Landa, 2014; Yerys et al., 2009). Interestingly, as the DSM changes removed the guidance that ADHD should not be diagnosed in children with an autism diagnosis when the children were aged 13 (2013), the group of children with ADHD and autism may be underrepresented in this study (Leitner, 2014).

Children with higher cognitive ability in this study were less likely to have ADHD recognised, perhaps as they are more able to mask their difficulties although they are still likely to be underperforming relative to their ability. This is supported by literature suggesting that those with a higher cognitive ability may have their ADHD‐related cognitive challenges overlooked (Cadenas et al., 2020). Most of our results were robust to sensitivity testing, however moving 11‐year‐olds into the early recognition group resulted in no difference in cognitive ability, which suggests that this factor's impact on ADHD recognition may be age dependent. Sadly, later measures of cognitive ability were unavailable in this dataset so we could not explore this further. The second sensitivity test, the exclusion of children with no diagnostic data on ADHD at age 5 and 7, resulted in an association of an autism diagnosis, or a parent with depression/anxiety being with earlier recognised ADHD, emphasising their importance in recognition, particularly in childhood.

Children with unrecognised ADHD had higher reported levels of physical activity than children with recognised ADHD. There is evidence that physical activity can improve some of the core ADHD symptoms, and improve executive function (Cerrillo‐Urbina et al., 2015; Jiang et al., 2022; Lambez et al., 2020; Mehren et al., 2020; Sun et al., 2022). The greater levels of physical activity that these children engaged with may have supported their management of ADHD symptoms, however, it should be recognised that more ADHD symptoms may prevent a child from coping with or being accepted into sports activities and clubs.

Others have also reported the association between parental depression/anxiety and offspring ADHD that we detected (Robinson et al., 2022). Parental ADHD must also be considered, as anxiety, depression and ADHD are correlated (Gnanavel et al., 2019; Katzman et al., 2017). Additionally, parental anxiety/depression may increase involvement with services that may identify ADHD in the children, or increase openness to seeking help for their child with ADHD symptoms with research suggesting that parental mental health is independently associated with being more likely to use ADHD related services (Sayal et al., 2015).

Given that ADHD diagnosis is often delayed in females, the results were stratified by sex where possible and results compared to explore sex‐specific factors. The only factor indicating a sex difference was emotional dysregulation, where males with recognised ADHD were more likely to have a higher score of emotional dysregulation than unrecognised ADHD males, but there was no difference in females. Emotional dysregulation is gaining recognition as a key aspect of ADHD symptomology, despite not being included in diagnostic criteria (Astenvald et al., 2022; Shaw et al., 2014; Soler‐Gutiérrez et al., 2023). If included in the criteria, females may be more likely to be recognised, as post‐hoc analysis revealed that females with both recognised and unrecognised ADHD were more likely to have higher levels of emotional dysregulation than females with no ADHD.

Emotional dysregulation has been linked to poorer education and mental health conditions in people with ADHD (Antony et al., 2022; Eyre et al., 2019; Qian et al., 2016). Due to the associated negative outcomes of emotional dysregulation, ADHD assessment is important, especially considering that females are at higher risk of mental health conditions generally, and within those with ADHD (Ottosen et al., 2019; Riecher‐Rössler, 2017). A consideration when examining the difference in males is the stereotype of emotional expression as a ‘feminine trait’, therefore the expression of emotions, particularly when dysregulated, may be more likely to be noticed in males (Barrett & Bliss‐Moreau, 2009). Additionally, males and females may act out their emotional dysregulation differently, adding to the complex interaction web of stereotypes and gender involved in ADHD referral and diagnosis. Emotional dysregulation alone does not explain this sex diagnosis bias. As these data were collected between 2007 and 2014, it is likely, that the stereotypes and assumptions that ADHD is a ‘male condition’ may have been more pertinent, whereas more recent clinical data may reflect that clinicians may be more aware of the condition in females (Young et al., 2020).

Two factors did not differ between any comparisons, which was inconsistent with the study hypotheses: maternal higher education and hobby frequency. The literature shows that there is a link between lower parental education level, and childhood ADHD symptoms (Torvik et al., 2020). All the groups compared had ADHD symptoms, so it may be this variable is related to general ADHD symptoms, but not whether they are recognised. The second factor, hobby frequency, aimed to capture non‐stereotypical hyperfocus fixations. The reason no differences were found may be due to the nature of the questions focussing on how often children did the activity, rather than the time they spent on it, or whether they had difficulty ceasing the activity or transferring attention to other tasks, alike those on the Adult Hyperfocus Questionnaire (Hupfeld et al., 2019).

Finally, we were not able to explore whether late‐onset ADHD explains why some are receiving ADHD diagnoses later in life, despite great interest in this debate, because the oldest children were aged 14, which is within the typically considered age range for ADHD onset (Riglin et al., 2022; Taylor et al., 2019).

The study strengths include the use of a population‐based sample which allowed comparisons between those with and without a clinical diagnosis of ADHD. Another benefit of using the MCS sample was the oversampling of typically underrepresented groups, for example, those who were socio‐economically disadvantaged or an ethnic minority, allowing appropriate representation (Joshi & Fitzsimons, 2016). Additionally, we applied sensitivity analyses testing for misclassification and missing data, confirming robustness and increasing confidence in the results. A Youth Advisory Group was consulted during the process of the research to ensure that it was relevant to the lived experiences of the neurodiverse community.

Limitations include missing data; drop‐outs are expected in any longitudinal study. Despite the loss of more children with than without ADHD, in a systematic study of retention in an earlier birth cohort, associations with predictors were unchanged, so loss to follow up may not have biased our results (Wolke et al., 2009). This was despite greater loss of families facing greater deprivation, it has been shown that the parents of children lost to follow up were more likely to have lower parental education, to not own the house they live in, be below the poverty line and have a lower age at birth (Wolke et al., 2009). We lacked data about the exact age at ADHD diagnosis as well as a lack of any ADHD diagnosis data past age 14, and the SDQ provided only a broad index of probable ADHD. Thus, some of those defined as unrecognised ADHD may not fulfil ADHD diagnostic criteria. Data on older ages may hold key information due to events that occur at 14–17, for example, school exams, that may trigger an ADHD referral, due to increased academic pressures on the young person. Additionally details of prior diagnoses, like anxiety or depression, may reveal why a diagnosis was overlooked, as found by others (Taylor et al., 2019). Parental ADHD symptoms may have been influential, and linked to the parental depression/anxiety factor, but were unfortunately not recorded. Additionally, we assumed that parental‐reports of diagnosed ADHD were correct, as we lacked reports from clinical records or diagnostic assessments, and we cannot assess how thorough and rigorous any clinical assessment was or who made the diagnosis. This may explain why the reported levels of ADHD diagnosis in the sample (5.2%) were at the higher end of the range of population prevalence estimates. This study design is observational, and therefore cannot disentangle whether observations are due to genetic influences, or other unmeasured confounders. Other studies with different designs are required to explore this (Thapar & Rutter, 2019). Lastly, the traits featured within these factors may not be exclusive to ADHD, however they were all chosen after careful consideration as to what may be associated with ADHD recognition (Cadenas et al., 2020; Cerrillo‐Urbina et al., 2015; Felt et al., 2014; Taylor et al., 2019).

Future research using a bigger sample of females is needed to replicate and expand on these findings and allow more comparisons across sex. Additionally, this research should feed into ADHD awareness policy for the general public–making health care professionals, teachers and parents aware of the factors that may delay recognition and diagnosis.

## CONCLUSION

These results suggest that children with more emotional and behavioural difficulties, emotional dysregulation, lower cognitive ability and poorer prosocial skills were more likely to be diagnosed earlier, and are overall more likely to be recognised if they, in addition, have a co‐occurring autism diagnosis, a parent with depression/anxiety and are less involved in sports clubs/activities. Co‐occurring autism diagnosis was the only differentiating factor between those recognised and those unrecognised with both ADHD symptoms and impact. When comparing sexes, higher emotional dysregulation is a key factor for recognition of ADHD in males, but for females, more emotional dysregulation does not appear to improve the likelihood of diagnosis. An alternative explanation is that these factors may protect against impact therefore delaying the diagnosis.

Overall, our findings suggest that children may have their ADHD missed, or diagnosed later if they are not particularly disruptive, are more cognitively able, and have better prosocial skills. This highlights the need to assess for the possibility of ADHD, regardless of academic and social abilities, if children are displaying symptoms, especially if they also have functional impact. Additionally, ADHD symptoms not currently in diagnostic criteria, for example, emotional dysregulation, should be considered to improve gender‐inclusive recognition of ADHD.

## AUTHOR CONTRIBUTIONS

**Isabella Barclay**: Conceptualization; data curation; formal analysis; investigation; methodology; visualization; writing—original draft. **Kapil Sayal**: Writing—review & editing. **Tamsin Ford**: Writing—review & editing. **Ann John**: Writing—review & editing. **Mark J. Taylor**: Supervision; writing—review & editing. **Anita Thapar:** Supervision; writing—review & editing. **Kate Langley**: Supervision; writing—review & editing. **Joanna Martin**: Conceptualization; funding acquisition; supervision; writing—review & editing.

## CONFLICT OF INTEREST STATEMENT

Tamsin Ford's research group receives funding for research methodology consultation from Place2Be and she is on the Board of the Association of Child and Adolescent Mental Health. Kate Langley has been on the ADHD scientific advisory board for Medice and received a speaker fee on a topic unrelated to this research. All other authors report no conflicts of interest.

## ETHICAL CONSIDERATIONS

The ethical approval for this specific study was granted by the Cardiff University School of Medicine Research Ethics Committee (reference SMREC 22/48). Data were provided with written parental informed consent, and then at age 11 the child also provided assent.

## Supporting information

Supporting Information S1

## Data Availability

The MCS data is in an open access and public repository and can be accessed on request via the UK Data Service: https://beta.ukdataservice.ac.uk/datacatalogue/series/series?id=2000031.

## ENDNOTE

^1^
It is important to note that sex and gender are different. Sex is most often defined by a health care professional examining the genitals of the neonate and assigning male or female. Sex is often treated as binary, but in some cases an individual can be intersex. The focus of this study will be sex assigned at birth (male or female), due to availability of data.
