# Supplementary material for: Investigating the reasons behind a later or missed diagnosis of attention‐deficit/hyperactivity disorder in young people: A population cohort study
Source: JCPP Adv. 2024 Dec 18;5(3):e12301. doi: 10.1002/jcv2.12301 (PMC12446718; doi:10.1002/jcv2.12301)
Supplement: Supplementary file 1 — Supporting Information S1 [file JCV2-5-e12301-s001.docx]

**Supporting Information**

**Table S1. Comparison of earlier recognised ADHD vs later recognised ADHD**

**Table S2. Comparison of those with recognised ADHD vs those with unrecognised ADHD.**

**Table S3. Comparisons of males with recognised ADHD vs males with unrecognised ADHD.**

**Table S4. Comparisons of females with recognised ADHD vs females with unrecognised ADHD.**

**Table S5. Interaction analysis using the results of the recognised males vs unrecognised males, and recognised females vs unrecognised females.**

**Table S6. Comparison of those with recognised ADHD vs those with unrecognised ADHD and impact.**

**Table S7. Sensitivity test with those with missing ADHD data at and 5 and 7 removed, before then comparing those with earlier recognised ADHD vs later recognised ADHD.**

**Table S8. Sensitivity test where the earlier vs later recognised ADHD groups were redefined, where the groups were earlier (age 5, 7 and 11) vs later (age 14).**

**Table S1. Comparison of earlier recognised ADHD vs later recognised ADHD.**

| **Variable** | **OR** | **Lower CI** | **Upper CI** | **p-value** | **Adjusted p-value** |
| --- | --- | --- | --- | --- | --- |
| **Cognitive Ability** | 1.20 | 1.04 | 1.38 | 0.013 | 0.019 |
| **Parental Depression/Anxiety** | 0.69 | 0.46 | 1.04 | 0.077 | 0.102 |
| **Maternal Higher Education** | 0.93 | 0.57 | 1.51 | 0.757 | 0.757 |
| **Autism Diagnosis** | 0.71 | 0.48 | 1.07 | 0.099 | 0.122 |
| **Hobby Frequency** | 0.85 | 0.58 | 1.25 | 0.411 | 0.469 |
| **Physical Activity** | 1.05 | 0.91 | 1.20 | 0.495 | 0.528 |
| **Emotional Dysregulation Age 5** | 0.27 | 0.18 | 0.41 | 6.00E-10 | 9.10E-09 |
| **Emotional Dysregulation Age 7** | 0.29 | 0.19 | 0.44 | 1.17E-08 | 9.39E-08 |
| **Prosocial Age 5** | 1.20 | 1.10 | 1.31 | 2.75E-05 | 6.29E-05 |
| **Prosocial Age 7** | 1.20 | 1.10 | 1.32 | 7.39E-05 | 1.48E-04 |
| **Peer Problems Age 5** | 0.77 | 0.70 | 0.85 | 2.00E-07 | 9.42E-07 |
| **Peer Problems Age 7** | 0.86 | 0.79 | 0.93 | 3.58E-04 | 0.001 |
| **Conduct Problems Age 5** | 0.78 | 0.71 | 0.86 | 2.35E-07 | 9.42E-07 |
| **Conduct Problems Age 7** | 0.81 | 0.74 | 0.88 | 2.00E-06 | 6.40E-06 |
| **Emotional Problems Age 5** | 0.84 | 0.76 | 0.92 | 1.20E-04 | 2.13E-04 |
| **Emotional Problems Age 7** | 0.84 | 0.78 | 0.91 | 1.96E-05 | 5.22E-05 |

Variables were explored to understand their relationship with the timing of ADHD diagnosis. With exact age as a covariate, and after false discovery rate (FDR) correction for multiple comparisons. OR =odds ratio, CI = confidence interval. Where earlier recognised ADHD is coded as 0, and later recognised ADHD is coded as 1.

**Table S2. Comparison of those with recognised ADHD vs those with unrecognised ADHD.**

| **Variable** | **OR** | **Lower CI** | **Upper CI** | **p-value** | **Adjusted p-value** |
| --- | --- | --- | --- | --- | --- |
| **Cognitive Ability** | 1.31 | 1.19 | 1.43 | 9.40E-09 | 1.88E-08 |
| **Parental Depression/Anxiety** | 0.47 | 0.37 | 0.59 | 1.86E-10 | 4.97E-10 |
| **Maternal Higher Education** | 1.32 | 1.00 | 1.75 | 0.053 | 0.054 |
| **Autism Diagnosis** | 0.11 | 0.09 | 0.15 | 3.10E-55 | 4.95E-54 |
| **Hobby Frequency** | 0.80 | 0.64 | 1.00 | 0.054 | 0.054 |
| **Physical Activity** | 1.10 | 1.00 | 1.20 | 0.042 | 0.048 |
| **Emotional Dysregulation Age 5** | 0.58 | 0.46 | 0.74 | 1.41E-05 | 2.25E-05 |
| **Emotional Dysregulation Age 7** | 0.40 | 0.31 | 0.52 | 9.07E-12 | 3.63E-11 |
| **Prosocial Age 5** | 1.09 | 1.04 | 1.15 | 0.001 | 0.001 |
| **Prosocial Age 7** | 1.13 | 1.07 | 1.19 | 8.17E-06 | 1.45E-05 |
| **Peer Problems Age 5** | 0.83 | 0.79 | 0.88 | 1.34E-10 | 4.30E-10 |
| **Peer Problems Age 7** | 0.81 | 0.77 | 0.85 | 4.33E-15 | 3.46E-14 |
| **Conduct Problems Age 5** | 0.84 | 0.79 | 0.89 | 4.39E-09 | 1.00E-08 |
| **Conduct Problems Age 7** | 0.81 | 0.76 | 0.85 | 9.25E-14 | 4.94E-13 |
| **Emotional Problems Age 5** | 0.93 | 0.88 | 0.98 | 0.008 | 0.010 |
| **Emotional Problems Age 7** | 0.91 | 0.87 | 0.95 | 0.000 | 1.69E-04 |

Variables were explored to understand their relationship with the likelihood of ADHD diagnosis. With exact age as a covariate, and after false discovery rate (FDR) correction for multiple comparisons. OR =odds ratio, CI = confidence interval. Where recognised ADHD is coded as 0, and unrecognised ADHD is coded as 1.

**Table S3. Comparisons of females with recognised ADHD vs females with unrecognised ADHD.**

| **Variable** | **OR** | **Lower CI** | **Upper CI** | **p-value** | **Adjusted p-value** |
| --- | --- | --- | --- | --- | --- |
| **Cognitive Ability** | 1.16 | 0.95 | 1.43 | 0.148 | 0.236 |
| **Parental Depression/Anxiety** | 0.60 | 0.37 | 0.97 | 0.036 | 0.115 |
| **Maternal Higher Education** | 0.92 | 0.54 | 1.58 | 0.771 | 0.786 |
| **Autism Diagnosis** | 0.16 | 0.09 | 0.28 | 8.93E-10 | 1.43E-08 |
| **Hobby Frequency** | 0.55 | 0.33 | 0.92 | 0.021 | 0.086 |
| **Physical Activity** | 1.16 | 0.95 | 1.42 | 0.135 | 0.236 |
| **Emotional Dysregulation Age 5** | 1.15 | 0.70 | 1.88 | 0.585 | 0.669 |
| **Emotional Dysregulation Age 7** | 0.70 | 0.42 | 1.17 | 0.174 | 0.237 |
| **Prosocial Age 5** | 1.06 | 0.94 | 1.19 | 0.327 | 0.402 |
| **Prosocial Age 7** | 1.12 | 0.99 | 1.25 | 0.064 | 0.172 |
| **Peer Problems Age 5** | 0.86 | 0.76 | 0.97 | 0.014 | 0.076 |
| **Peer Problems Age 7** | 0.81 | 0.73 | 0.91 | 2.74E-04 | 0.002 |
| **Conduct Problems Age 5** | 0.91 | 0.80 | 1.03 | 0.128 | 0.236 |
| **Conduct Problems Age 7** | 0.90 | 0.80 | 1.02 | 0.091 | 0.208 |
| **Emotional Problems Age 5** | 1.02 | 0.90 | 1.14 | 0.786 | 0.786 |
| **Emotional Problems Age 7** | 0.94 | 0.85 | 1.03 | 0.177 | 0.237 |

Variables were explored to understand their relationship with the likelihood of ADHD diagnosis, split by sex to allow analysis in females specifically. With exact age as a covariate, and after false discovery rate (FDR) correction for multiple comparisons. OR =odds ratio, CI = confidence interval. Where recognised ADHD is coded as 0, and unrecognised ADHD is coded as 1.

**Table S4. Comparisons of males with recognised ADHD vs males with unrecognised ADHD.**

| **Variable** | **OR** | **Lower CI** | **Upper CI** | **p-value** | **Adjusted p-value** |
| --- | --- | --- | --- | --- | --- |
| **Cognitive Ability** | 1.30 | 1.17 | 1.45 | 6.20E-07 | 1.24E-06 |
| **Parental Depression/Anxiety** | 0.41 | 0.31 | 0.54 | 2.67E-10 | 8.56E-10 |
| **Maternal Higher Education** | 1.54 | 1.10 | 2.15 | 0.011 | 0.0136 |
| **Autism Diagnosis** | 0.12 | 0.09 | 0.16 | 5.91E-41 | 9.46E-40 |
| **Hobby Frequency** | 0.77 | 0.59 | 1.00 | 0.050 | 0.0538 |
| **Physical Activity** | 1.10 | 1.00 | 1.22 | 0.061 | 0.0607 |
| **Emotional Dysregulation Age 5** | 0.50 | 0.38 | 0.67 | 2.92E-06 | 5.20E-06 |
| **Emotional Dysregulation Age 7** | 0.35 | 0.26 | 0.48 | 6.52E-11 | 2.61E-10 |
| **Prosocial Age 5** | 1.07 | 1.01 | 1.14 | 0.024 | 0.0280 |
| **Prosocial Age 7** | 1.09 | 1.03 | 1.16 | 0.004 | 0.0056 |
| **Peer Problems Age 5** | 0.83 | 0.78 | 0.89 | 4.67E-08 | 1.25E-07 |
| **Peer Problems Age 7** | 0.81 | 0.76 | 0.86 | 1.40E-11 | 7.49E-11 |
| **Conduct Problems Age 5** | 0.83 | 0.78 | 0.89 | 1.48E-07 | 3.39E-07 |
| **Conduct Problems Age 7** | 0.79 | 0.74 | 0.85 | 4.52E-12 | 3.62E-11 |
| **Emotional Problems Age 5** | 0.91 | 0.85 | 0.96 | 0.001 | 0.0020 |
| **Emotional Problems Age 7** | 0.89 | 0.85 | 0.95 | 9.21E-05 | 0.0001 |

Variables were explored to understand their relationship with the likelihood of ADHD diagnosis, split by sex to allow analysis in males specifically. With exact age as a covariate, and after false discovery rate (FDR) correction for multiple comparisons. OR =odds ratio, CI = confidence interval. Where recognised ADHD is coded as 0, and unrecognised ADHD is coded as 1.

**Table S5. Interaction analysis using the results of the recognised males vs unrecognised males, and recognised females vs unrecognised females.**

| **Variable** | **OR** | **Lower CI** | **Upper CI** | **p-value** |
| --- | --- | --- | --- | --- |
| **Cognitive Ability** | 0.90 | 0.72 | 1.13 | 0.368 |
| **Parental Depression/Anxiety** | 1.45 | 0.84 | 2.52 | 0.183 |
| **Maternal Higher Education** | 0.59 | 0.32 | 1.12 | 0.108 |
| **Autism Diagnosis** | 1.32 | 0.68 | 2.58 | 0.416 |
| **Hobby Frequency** | 0.72 | 0.41 | 1.28 | 0.262 |
| **Physical Activity** | 1.05 | 0.84 | 1.31 | 0.658 |
| **Emotional Dysregulation Age 5** | 2.27 | 1.28 | 4.02 | 0.005 |
| **Emotional Dysregulation Age 7** | 1.97 | 1.08 | 3.58 | 0.027 |
| **Prosocial Age 5** | 0.99 | 0.87 | 1.13 | 0.849 |
| **Prosocial Age 7** | 1.02 | 0.89 | 1.16 | 0.780 |
| **Peer Problems Age 5** | 1.03 | 0.90 | 1.18 | 0.646 |
| **Peer Problems Age 7** | 1.02 | 0.90 | 1.16 | 0.784 |
| **Conduct Problems Age 5** | 1.09 | 0.94 | 1.26 | 0.262 |
| **Conduct Problems Age 7** | 1.14 | 1.00 | 1.31 | 0.054 |
| **Emotional Problems Age 5** | 1.12 | 0.98 | 1.28 | 0.085 |
| **Emotional Problems Age 7** | 1.05 | 0.93 | 1.17 | 0.430 |

Variables were explored to understand their relationship with the likelihood of ADHD diagnosis, here the results of the male and female split analysis were compared in order to analyse differences and similarities. With exact age as a covariate, and after false discovery rate (FDR) correction for multiple comparisons. OR =odds ratio, CI = confidence interval. Where recognised ADHD males vs unrecognised ADHD males is coded as 0, and recognised ADHD females vs unrecognised ADHD females is coded as 1.

**Table S6. Comparison of those with recognised ADHD vs those with unrecognised ADHD and impact.**

| **Variable** | **OR** | **Lower CI** | **Upper CI** | **p-value** | **Adjusted p-value** |
| --- | --- | --- | --- | --- | --- |
| **Cognitive Ability** | 1.02 | 0.90 | 1.16 | 0.729 | 0.922 |
| **Parental Depression/Anxiety** | 0.80 | 0.56 | 1.13 | 0.208 | 0.310 |
| **Maternal Higher Education** | 1.02 | 0.67 | 1.57 | 0.912 | 0.930 |
| **Autism Diagnosis** | 0.34 | 0.24 | 0.49 | 6.43E-09 | 1.03E-07 |
| **Hobby Frequency** | 0.74 | 0.53 | 1.05 | 0.088 | 0.236 |
| **Physical Activity** | 1.01 | 0.89 | 1.14 | 0.887 | 0.930 |
| **Emotional Dysregulation Age 5** | 1.48 | 1.03 | 2.12 | 0.035 | 0.112 |
| **Emotional Dysregulation Age 7** | 1.59 | 1.09 | 2.33 | 0.017 | 0.081 |
| **Prosocial Age 5** | 0.96 | 0.89 | 1.03 | 0.213 | 0.310 |
| **Prosocial Age 7** | 0.92 | 0.85 | 0.99 | 0.020 | 0.081 |
| **Peer Problems Age 5** | 1.05 | 0.97 | 1.13 | 0.209 | 0.310 |
| **Peer Problems Age 7** | 1.06 | 0.98 | 1.13 | 0.132 | 0.263 |
| **Conduct Problems Age 5** | 1.00 | 0.92 | 1.08 | 0.930 | 0.930 |
| **Conduct Problems Age 7** | 1.01 | 0.94 | 1.09 | 0.749 | 0.922 |
| **Emotional Problems Age 5** | 1.06 | 0.98 | 1.14 | 0.130 | 0.263 |
| **Emotional Problems Age 7** | 1.09 | 1.02 | 1.16 | 0.014 | 0.081 |

Variables were explored to understand their relationship with the likelihood of ADHD diagnosis. With exact age as a covariate, and after false discovery rate (FDR) correction for multiple comparisons. OR =odds ratio, CI = confidence interval. Where recognised ADHD is coded as 0, and unrecognised ADHD and impact is coded as 1.

**Table S7. Sensitivity test with those with missing ADHD data at and 5 and 7 removed, before then comparing those with earlier recognised ADHD vs later recognised ADHD.**

| **Variable** | **OR** | **Lower CI** | **Upper CI** | **p-value** | **Adjusted p-value** |
| --- | --- | --- | --- | --- | --- |
| **Cognitive Ability** | 1.21 | 1.05 | 1.40 | 0.009 | 0.013 |
| **Parental Depression/Anxiety** | 0.63 | 0.42 | 0.95 | 0.027 | 0.036 |
| **Maternal Higher Education** | 0.93 | 0.57 | 1.52 | 0.776 | 0.776 |
| **Autism Diagnosis** | 0.66 | 0.44 | 0.98 | 0.040 | 0.050 |
| **Hobby Frequency** | 0.83 | 0.56 | 1.23 | 0.359 | 0.410 |
| **Physical Activity** | 1.05 | 0.92 | 1.21 | 0.472 | 0.503 |
| **Emotional Dysregulation Age 5** | 0.27 | 0.18 | 0.41 | 6.00E-10 | 9.10E-09 |
| **Emotional Dysregulation Age 7** | 0.28 | 0.18 | 0.44 | 9.90E-09 | 7.89E-08 |
| **Prosocial Age 5** | 1.20 | 1.10 | 1.31 | 2.75E-05 | 6.29E-05 |
| **Prosocial Age 7** | 1.20 | 1.10 | 1.32 | 6.55E-05 | 1.31E-04 |
| **Peer Problems Age 5** | 0.77 | 0.70 | 0.85 | 2.00E-07 | 9.42E-07 |
| **Peer Problems Age 7** | 0.86 | 0.79 | 0.93 | 2.99E-04 | 4.79E-04 |
| **Conduct Problems Age 5** | 0.78 | 0.71 | 0.86 | 2.35E-07 | 9.42E-07 |
| **Conduct Problems Age 7** | 0.80 | 0.74 | 0.88 | 1.50E-06 | 4.79E-06 |
| **Emotional Problems Age 5** | 0.84 | 0.76 | 0.92 | 1.20E-04 | 2.13E-04 |
| **Emotional Problems Age 7** | 0.84 | 0.78 | 0.91 | 1.92E-05 | 5.13E-05 |

A sensitivity test to understand if missing data had a significant impact on the results. With exact age as a covariate, and after false discovery rate (FDR) correction for multiple comparisons. OR =odds ratio, CI = confidence interval. Where earlier recognised ADHD is coded as 0, and later recognised ADHD is coded as 1.

**Table S8. Sensitivity test where the earlier vs later recognised ADHD groups were redefined, where the groups were earlier (age 5, 7 and 11) (coded as 0) vs later (age 14) (coded as 1).**

| **Variable** | **OR** | **Lower CI** | **Upper CI** | **p-value** | **Adjusted p-value** |
| --- | --- | --- | --- | --- | --- |
| **Cognitive Ability** | 1.17 | 0.97 | 1.42 | 0.102 | 0.136 |
| **Parental Depression/Anxiety** | 0.90 | 0.54 | 1.49 | 0.673 | 0.828 |
| **Maternal Higher Education** | 1.02 | 0.56 | 1.87 | 0.943 | 0.943 |
| **Autism Diagnosis** | 0.45 | 0.28 | 0.74 | 0.002 | 0.007 |
| **Hobby Frequency** | 0.95 | 0.59 | 1.55 | 0.847 | 0.903 |
| **Physical Activity** | 1.02 | 0.86 | 1.21 | 0.829 | 0.903 |
| **Emotional Dysregulation Age 5** | 0.39 | 0.24 | 0.64 | 1.68E-04 | 0.001 |
| **Emotional Dysregulation Age 7** | 0.38 | 0.24 | 0.61 | 7.56E-05 | 0.001 |
| **Prosocial Age 7** | 1.18 | 1.04 | 1.33 | 0.009 | 0.015 |
| **Prosocial Age 5** | 1.18 | 1.05 | 1.33 | 0.007 | 0.014 |
| **Peer Problems Age 5** | 0.79 | 0.69 | 0.90 | 0.001 | 0.003 |
| **Peer Problems Age 7** | 0.86 | 0.77 | 0.96 | 0.007 | 0.014 |
| **Conduct Problems Age 5** | 0.88 | 0.78 | 0.99 | 0.029 | 0.042 |
| **Conduct Problems Age 7** | 0.84 | 0.75 | 0.94 | 0.003 | 0.009 |
| **Emotional Problems Age 5** | 0.85 | 0.74 | 0.96 | 0.012 | 0.018 |
| **Emotional Problems Age 7** | 0.84 | 0.76 | 0.94 | 0.002 | 0.007 |

A sensitivity test to understand if the definition of the earlier and later recognised ADHD groups had a significant impact on the results. With exact age as a covariate, and after false discovery rate (FDR) correction for multiple comparisons. OR =odds ratio, CI = confidence interval. Where earlier recognised ADHD is coded as 0, and later recognised ADHD is coded as 1.
